# Portuguese translation, cultural adaptation and psychometric properties of the temporomandibular joint scale: a cross-sectional study

**DOI:** 10.1007/s10006-024-01300-8

**Published:** 2024-10-31

**Authors:** Mariana Cervaens, Jéssica Pereira, André Magalhães, Mário Esteves, Rui Vilarinho, Verónica Abreu, Luísa Amaral

**Affiliations:** 1 FP-I3ID, FP-BHS, Escola Superior de Saúde Fernando Pessoa, Porto, Portugal; 2 RISE-HEALTH, Universidade Fernando Pessoa, Porto, Portugal; 3 Escola Superior de Saúde Fernando Pessoa, Porto, Portugal; 4 Centro de Investigação em Reabilitação (CIR), Escola Superior de Saúde, Instituto Politécnico do Porto, Porto, Portugal; 5 Insight Piaget Research Centre for Ecological Human Development, Piaget Institute, Lisboa, Portugal

**Keywords:** Temporomandibular joint Scale, translation, cultural adaptation, reliability, validity

## Abstract

**Background:**

The temporomandibular joint (TMJ) scale assesses the severity of temporomandibular joint disorders (TMD), yet a European Portuguese translation is lacking.

**Objectives:**

To translate, cross-culturally adapt and to examine the psychometric properties (construct validity and reliability) of the TMJ scale.

**Methods:**

Translation and cultural adaptation were carried out according to international recommendations, including initial translation, evaluation of this translation and cultural adaptation by a panel of experts, and back translation. The final Portuguese version was used to examine the reliability and validity, and participants with TMD were recruited from a Portuguese outpatient clinic. Reliability measures included internal consistency with Cronbach’s alpha and test-retest reliability with the intraclass correlation coefficient (ICC2,1). The Spearman correlation comparing the TMJ scale with the Fonseca and Helkimo indexes was used to assess the construct validity.

**Results:**

A total of 63 participants (23 ± 2 years; 61,9% female) were included. Similar internal consistency was observed between the two moments of application (0.921 and 0.918), and test-retest reliability was excellent, with an ICC2,1 = 0.998 (95%CI: 0.988–0.999). Robust positive correlations (rho 0.554–0.611, *p* < 0.001) were found between the TMJ scale and Fonseca and Helkimo indexes.

**Conclusion:**

The European Portuguese version of TMJ scale is now available to improve the assessment of severity of TMD in routine clinical practice. This version is also reliable and valid.

**Supplementary Information:**

The online version contains supplementary material available at 10.1007/s10006-024-01300-8.

## Introduction

The temporomandibular joint (TMJ) has a crucial role in several physiological functions, including chewing, speech, breathing and facial expression [1–3]. The intricate structure TMJ involves the articulation of the temporal bone and the mandible, encompassing a complex interplay of muscles, ligaments, and a fibrocartilaginous disc [4]. Its unique biomechanics, which includes both hinge and glide movements, allow for a wide range of jaw motions [5]. However, this finely tuned system is susceptible to malfunctions that leads to temporomandibular disorders (TMD). These disorders are defined as a collection of signs and symptoms affecting the masticatory system, characterised by pain, limited jaw movement, and dysfunction of the TMJ [6]. Epidemiologic data indicates that the prevalence of TMD symptoms is approximately 30% in adults and 5–15% in children, with a potential increase during adolescence [2, 7]. Women are two to three times more susceptible to TMD than men [7].

The aetiology of TMD is complex and multifactorial, and while internal derangements within the joint, including disc displacement and osteoarthritis, have been implicated in the past [8], muscular factors such as myofascial pain and masticatory dysfunction may play an important role [7]. Other factors such as stress [9], genetic predisposition [10] hormonal fluctuations [11] can act as potential triggers or aggravate existing conditions. Patient reported outcome measures (e.g., scales and questionnaires) are important in the assessment of TMD [12]. Typically, these instruments offer insight into the subjective experiences that include pain intensity, functional limitations, psychosocial impact and quality of life [13, 14]. The TMJ scale, developed by the Pain Resource Center in the United States of America (USA) [15], has been used to assess the presence and severity of TMD symptoms [16–18]. This scale consists of 97 items, using a diagram and a four-point rating scale [17]. The TMJ Scale is not available in Portuguese, which limits its use in Portugal. Therefore, this study aimed to translate, cross-culturally adapt the European Portuguese version of the TMJ scale. Another aim was to examine the reliability and construct validity of the European version of the scale.

## Methodology

The TMJ Scale was translated from English and culturally adapted for the Portuguese population after written consent from the authors of the original scale [15]. The cross-cultural adaptation process was carried out according to the current guidelines and divided in 5 stages [19]. For Stage 1 - translation, a bilingual healthcare professional and a bilingual translator independently performed a forward translation of the TMJ Scale from English to Portuguese. When translators experienced difficulty to reach consensus, the English version was used to assist [20]. The 2 Portuguese versions were then synthesised into 1 version in a consensus meeting (Stage 2 - synthesis). The discrepancies between versions were resolved by a third party [21] who performed a written report detailing the difficulties encountered and how they were resolved.

Two different bilingual translators, non-health professionals whose native language is English and who are fluent in Portuguese, performed two backward translations of the Portuguese TMJ Scale synthesis version, back into English (Stage 3 - back translation). Both translators were unaware of the original version of the TMJ [21]. Three specialists from the temporomandibular rehabilitation field, together with the four translators and the third party, involved in the project, reviewed all translated and back-translated versions to reach consensus on possible discrepancies. Then, a pre-final version of the TMJ Scale was produced (Stage 4 - expert committee review). The pre-final version of the TMJ Scale was applied to a panel of 63 individuals (Stage V - pretesting) whose sociodemographic and academic data was collected to characterise the sample [22, 23]. Each individual who completed the questionnaire was interviewed to assess the layout of the instrument, as well as the comprehension of the instructions and the different items, the receptiveness and adherence to its contents. For each participant, the total score (dependent variable) in the TMJ questionnaire was obtained.

The final version was then produced and distributed to the same panel. All questionnaire versions were printed and distributed for self-completion.

### Clinical trial number

not applicable.

### Study sample

The patient panel (for stage 5 - pretesting and final version testing) comprised individuals experiencing TMJ pain recruited in April 2019 from the outpatient clinic of Fernando Pessoa University (UFP). Participants were adults aged 18 years and older, excluding those with a history of significant craniofacial trauma, prior surgical TMJ interventions, or orofacial tumours.

### Reliability

Participants were asked to complete the TMJ scale twice in-person, 7 days apart. A well-trained physiotherapist conducted the in-person interviews. Participants and the physiotherapist were blinded to the answers/scores of the instrument on the first administration.

### Validity

The construct validity was performed correlating the two TMJ assessments with two other instruments, the Fonseca [24] and Helkimo [25] indexes (independent variables). Fonseca index [26] has a sensitivity of 94.23–98.21% and a specificity of 87.72%, whilst Helkimo index [27] shows a sensitivity of 86.67%, a specificity of 68.09% [27] predicting TMD.

### Statistical analysis

Continuous variables were characterised by their mean, minimum, and maximum values, while categorical variables were presented as absolute frequencies. The internal consistency was assessed using Cronbach’s alpha and a value higher than 0.70 was considered an acceptable level of consistency [28]. Relative reliability in test-retest was explored with the Intraclass Correlation Coefficient (ICC) Eqs. (1, 2) and the respective 95% confidence intervals (95%CI). The ICC was interpreted as excellent (> 0.75), moderate to good (0.4–0.75) or poor (< 0.4). The construct validity of the TMJ scale was assessed using the Spearman correlation coefficient (rho), comparing it to the Fonseca and Helkimo indexes. This coefficient was interpreted as strong (≥ 0.70), moderate (0.30–0.70) or weak (≤ 0.30) [29]. According to Consensus-based Standards for the selection of health Measurement Instruments (COSMIN) guidelines, a sample size of at least 50 participants are needed to test the psychometric properties of PROMs. Statistical analysis was performed using the Statistical Package for Social Sciences (SPSS), version 25.0 for Windows, with a significance level set at 5%.

## Results

The initial translation and synthesis, as well as the back-translation and synthesis, included statements from the translators and back-translators, respectively. The panel of experts’ modifications to the semantic, idiomatic, experiential, and conceptual aspects resulted in the final version of the scale. The reliability and validity sample consisted of 63 participants, with a mean age of 23 ± 2 years, ranging from 18 to 26 years. Most of the participants were female (*n* = 39), with a mean body mass index (BMI) of 23.7 ± 1.7 kg/m². Following pre-testing of the final version, it became evident that the items in the translated and adapted version were universally comprehensible and interpretable by all participants.

### Reliability

The Cronbach’s alpha test indicated strong and similar internal consistency at both time points, 0.921 at the first assessment and 0.918 at the second assessment. Item-by-item analysis showed consistent Cronbach’s alpha values, ranging from 0.917 to 0.924 in the first assessment and from 0.915 to 0.922 in the second assessment. Excellent test-retest reliability was found with an ICC of 0.998 (95% CI: 0.988–0.999). Total scores at both assessments of TMJ are reported in Table 1.

**Table 1:** Descriptive analysis of the total score at both moments

*n* = 63	Mean	Standard deviation	Minimum	Maximum
**Total Score at 1º moment**	105,37	36,291	49	217
**Total Score at 2º moment**	107,48	35,701	54	218

### Construct validity

The construct validity of the TMJ Scale was demonstrated by moderate correlations with the Fonseca Index at both the first (*r* = 0.554, *p* < 0.001) and second (*r* = 0.585, *p* < 0.001) assessment moments, and with the Helkimo Index at both the first (*r* = 0.611, *p* < 0.001) and second (*r* = 0.606, *p* < 0.001) assessment moments.

## Discussion

Our study shows that the European version of the TMJ scale is reliable and valid in people with TMD. The adaptation of an instrument is necessary when there are cultural differences between the instrument’s origin and the target population [30]. This process should involve a rigorous evaluation of both the cultural adaptation itself and the instrument’s psychometric properties [30]. In the literature, there are several TMD assessment instruments available [31, 32]. The Helkimo and Fonseca indexes are two of the most widely used assessment tools, especially in Portuguese countries [33, 34]. The Helkimo index has been widely used instrument in studies for symptom-based classification of TMD, mainly due to its ease of use in large-scale population research. Nevertheless, it has certain limitations, such as the potential to classify a person with mild TMD based only on three criteria for evaluating facial pain [31].

The Fonseca index is based on Helkimo and assesses the severity of the TMD based on its signs and symptoms [24]. This instrument is particularly useful for early stage TMD [35]. Both the Fonseca and Helkimo Indexes [24, 25] classify individuals with common symptoms like headaches or palpation tenderness as having TMD, regardless of the absence of definitive clinical signs. However, these symptoms can occur independently of TMD [33]. This binary approach might limit the accuracy of diagnoses, particularly when assessing individuals without obvious symptoms. The TMJ Scale facilitates a nuanced assessment by integrating symptomatic presentation with objective clinical indicators, thereby it may improve the diagnostic precision and ensure comprehensive evaluation across diverse patient demographics. Our research findings affirm the robustness of the adapted TMJ Scale, showcasing strong reliability, internal consistency, and construct validity for evaluating TMD among the Portuguese population.

This study assessed the reliability of the Portuguese TMJ Scale through test-retest reliability and internal consistency analysis. Test-retest reliability indicates the scale’s ability to produce consistent scores when administered to the same individuals on multiple occasions [36]. Internal consistency assesses how well items within the scale measure the same underlying construct [37]. Our data indicates a strong association between repeated administrations of the Portuguese TMJ Scale, supported by a very high ICC (0.998, 95% CI). To our knowledge, the TMJ Scale hasn’t been translated and culturally adapted to any other language. The original authors reported that the instrument was reasonably reliable and accurately screened for disorders in both patients and non-patients [16]. Regarding the other instruments, the Fonseca index has shown a high reliability and good correlation with Helkimo index [32, 34]. The reliability data available for the Helkimo Index is limited in terms of quality. Despite this, the Helkimo Index is valued in clinical settings for its simplicity in evaluating TMD [27, 36]. Using the Cronbach’s alpha coefficient, we observed excellent internal consistency across all domains, supported by a strong correlation among all scale items, which affirms its reliability. According to Maroco [36], decreased variability among items across subjects correlates with reduced measurement error, thus reinforcing the scale’s reliability. However, the inclusion of 97 questions in TMJ questionnaire may introduce potential biases, as lengthy questionnaires can inflate alpha values without necessarily enhancing internal consistency. The construct validity was assessed correlating the TMJ Scale with both the Fonseca and Helkimo Indexes. Construct validity is a measure of the extent of which scores on a particular instrument relate to other measures in a manner that is consistent with theoretically derived hypotheses concerning the concepts that are being measured [38, 39]. Our results revealed a moderate to strong association between all measures.

### Limitations

The present study has limitations that should be considered. Despite the consent to culturally adapt the “Temporomandibular Joint Scale”, our request to access the domains inherent in each question was denied by both the scale’s author and the Pain Resource Centre. Therefore, only the total/general scores were obtained, and not the domain-specific scores. Another limitation, similarly, to other studies, was the higher proportion of female individuals, possibly related to a higher prevalence of TMDs in the female population. Moreover, to our knowledge, the TMJ hasn’t been translated and culturally adapted to any other language, which limited the results comparison. In addition, we also recommend in further studies the comparison of the TMJ scale with the DC/TMD, as the gold standard measure to assess the TMD.

## Conclusion

The European Portuguese version of the TMJ scale is now available to improve the assessment of the severity of TMD in routine clinical practice. The results of this study also confirm that the TMJ is a reliable and valid PROMs. Furthermore, these results underline the potential of this scale for both clinical diagnosis and symptoms identification on TMD in the Portuguese population.

## Electronic Supplementary Material

Below is the link to the electronic supplementary material.

Supplementary Material 1

Supplementary Material 2

## Data Availability

No datasets were generated or analysed during the current study.

## References

[1] Manfredini D, Segù M, Arveda N et al (2016) <ArticleTitle Language="En">Temporomandibular Joint disorders in patients with different facial morphology. A systematic review of the literature. J Oral Maxillofac Surg 74(1):29–46. 10.1016/J.JOMS.2015.07.006 26255097 10.1016/j.joms.2015.07.006

[2] Ryan J, Hassan N, Hilton G, Wickham J, Akhter R, Ibaragi S (2019) Epidemiology of Temporomandibular Disorder in the General Population: a Systematic Review. Adv Dentistry Oral Health 10(3):1–13. 10.19080/ADOH.2019.10.555787

[3] Lund JP (1991) Mastication and its Control by the Brain Stem. Crit Reviews Oral Biology Med 2(1):33–64. 10.1177/10454411910020010401 10.1177/10454411910020010401 1912143

[4] Standring S (2015) Gray’s Anatomy - The Anatomical Basis of Clinical Practice, 41st edn. Elsevier

[5] Palla P S (2016) Anatomy and Pathophysiology of the Temporomandibular Joint. Funct Occlusion Restor Dentistry Prosthodont 2016:67–85. 10.1016/B978-0-7234-3809-0.00006-1

[6] American Academy of Orofacial Pain (2018) Orofacial Pain: Guidelines for Assessment, Diagnosis, and Management. 6th ed. (de Leeuw R, Klasser,Gary D., eds.). Quintessence Publishing

[7] Bueno CH, Pereira DD, Pattussi MP, Grossi PK, Grossi ML (2018) Gender differences in temporomandibular disorders in adult populational studies: A systematic review and meta-analysis. J Oral Rehabil 45(9):720–729. 10.1111/JOOR.12661 29851110 10.1111/joor.12661

[8] Jennings EA, Williams MC, Staikopoulos V, Ivanusic JJ (2012) Neurobiology of Temporomandibular Joint Pain: Therapeutic Implications. Semin Orthod 18(1):63–72. 10.1053/J.SODO.2011.10.003

[9] Staniszewski K, Lygre H, Bifulco E et al (2018) Temporomandibular Disorders Related to Stress and HPA-Axis Regulation. Pain Res Manag 2018(1):7020751. 10.1155/2018/7020751 29854038 10.1155/2018/7020751 PMC5954859

[10] Scariot R, Corso PFCL, Sebastiani AM, Vieira AR (2018) The many faces of genetic contributions to temporomandibular joint disorder: An updated review. Orthod Craniofac Res 21(4):186–201. 10.1111/OCR.12239 30204294 10.1111/ocr.12239

[11] Moreno AGUT, Bezerra AGV, Alves-Silva EG et al (2021) Influence of estrogen on pain modulation in temporomandibular disorder and its prevalence in females: an integrative review. Res Soc Dev 10(2):e38510212453–e38510212453. 10.33448/RSD-V10I2.12453

[12] Brown DT, Gaudet EL (1994) Outcome Measurement for Treated and Untreated TMD Patients Using the TMJ Scale. CRANIO^®^ 12(4):216–222. 10.1080/08869634.1994.11678024 7828202 10.1080/08869634.1994.11678024

[13] Durham J, Steele J, Moufti MA, Wassell R, Robinson P, Exley C (2011) Temporomandibular disorder patients’ journey through care. Community Dent Oral Epidemiol 39(6):532–541. 10.1111/j.1600-0528.2011.00608.x 21299587 10.1111/j.1600-0528.2011.00608.x

[14] Ohrbach R, Larsson P, List T (2008) The jaw functional limitation scale: development, reliability, and validity of 8-item and 20-item versions. J Orofac Pain 22(3):219–230 18780535

[15] Levitt SR, Lundeen TF, McKinney MW (1988) Initial studies of a new assessment method for temporomandibular joint disorders. J Prosthet Dent 59(4):490–495. 10.1016/0022-3913(88)90048-0 3162995 10.1016/0022-3913(88)90048-0

[16] Levitt SR, McKinney MW, Lundeen TF (1988) The TMJ Scale: Cross-Validation and Reliability Studies. CRANIO^®^ 6(1):17–25. 10.1080/08869634.1988.11678216 3162835 10.1080/08869634.1988.11678216

[17] Lundeen TF, Levitt SR, McKinney MW (1988) Evaluation of temporomandibular joint disorders by clinician ratings. J Prosthet Dent 59(2):202–211. 10.1016/0022-3913(88)90016-9 3422687 10.1016/0022-3913(88)90016-9

[18] Yamaguchi D, Motegi E, Nomura M et al (2002) Evaluation of Psychological Factors in Orthodontic Patients with TMD as applied to the TMJ Scale. Original Article 83 Bull Tokyo dent Coll 43(2):8387 10.2209/tdcpublication.43.83 12174668

[19] Beaton D, Bombardier C, Guillemin F, Ferraz M (2002) Recommendations for the cross-cultural adaptation of health status measures. Am Acad Orthop Surg Revis Published online 2002:1–9

[20] Mokkink Cecilia AC, Prinsen Donald L, Patrick Jordi Alonso Lex M, Bouter LB, Mokkink CL (2024) COSMIN Study Design checklist for Patient-reported outcome measurement instruments. Accessed July 2. https://www.cosmin.nl

[21] Ferreira L, Neves AN, Campana MB, Da M, Gomes C, Fernandes Tavares C (2014) Guia da AAOS/IWH: sugestões para adaptação transcultural de escalas 1. Avaliação Psicológica 13(3):457–461

[22] Pedro AR, Amaral O, Escoval A (2016) Literacia em saúde, dos dados à ação: tradução, validação e aplicação do European Health Literacy Survey em Portugal. Revista Portuguesa de Saúde Pública 34(3):259–275. 10.1016/J.RPSP.2016.07.002

[23] Duarte HMS, Sousa PML, Dixe M, dos ACR, Professora R (2017) Validação da versão portuguesa da escala de satisfação dos estudantes de enfermagem relativamente à Simulação de Alta-Fidelidade (ESEE-SAF). Instituto Politécnico de Leiria U de I em SES de S de L, ed. Construindo conhecimento em Enfermagem à pessoa em situação crítica. Published online 2017:185–196. Accessed July 3, 2024. https://iconline.ipleiria.pt/handle/10400.8/2889

[24] da Fonseca DM, Bonfante G, Valle AL, de do, Freitas SFT (1994) Diagnóstico pela anamnese da disfunção craniomandibular. RGO 42(1):23–28

[25] Helkimo M (1974) Studies on function and dysfunction of the masticatory system. II. Index for anamnestic and clinical dysfunction and occlusal state. Sven Tandlak Tidskr 67(2):101–121 4524733

[26] Yap AU, Zhang MJ, Lei J, Fu KY (2024) Accuracy of the Fonseca Anamnestic Index for identifying pain-related and/or intra-articular Temporomandibular Disorders. CRANIO^®^ 42(3):259–266. 10.1080/08869634.2021.1954375 34259594 10.1080/08869634.2021.1954375

[27] Alonso-Royo R, Sánchez-Torrelo CM, Ibáñez-Vera AJ et al (2021) Validity and Reliability of the Helkimo Clinical Dysfunction Index for the Diagnosis of Temporomandibular Disorders. Diagnostics 11(3):472. 10.3390/diagnostics11030472 33800185 10.3390/diagnostics11030472 PMC8000811

[28] Taber KS (2018) The Use of Cronbach’s Alpha When Developing and Reporting Research Instruments in Science Education. Res Sci Educ 48(6):1273–1296. 10.1007/s11165-016-9602-2

[29] Akoglu H (2018) User’s guide to correlation coefficients. Turk J Emerg Med 18(3):91–93. 10.1016/j.tjem.2018.08.001 30191186 10.1016/j.tjem.2018.08.001 PMC6107969

[30] Costa LMR da, de Medeiros DL, Ries LGK, Beretta A, de Noronha MA (2014) Assessment of cross-cultural adaptations and measurement properties of self-report outcome measures in Portuguese relevant to temporomandibular disorders: a systematic review. Fisioterapia e Pesquisa 21(2):107–112. 10.1590/1809-2950/35421022014

[31] Borges REA, Mendonça L, da Santos Calderon RA P (2024) Diagnostic and screening inventories for temporomandibular disorders: A systematic review. CRANIO^®^ 42(3):341–347. 10.1080/08869634.2021.1954376 34275426 10.1080/08869634.2021.1954376

[32] Kumar N, Daigavane P, Jain S, Mantri N (2022) Review of Various Clinical Assessment Indices and Orthodontic Management for Temporomandibular Joint Disorders. Cureus Published online Oct 19. 10.7759/cureus.30492 10.7759/cureus.30492 PMC9674566 36415405

[33] Chaves TC, de Oliveira AS, Grossi DB (2008) Principais instrumentos para avaliação da disfunção temporomandibular, parte I: índices e questionários; uma contribuição para a prática clínica e de pesquisa. Fisioterapia e Pesquisa 15(1):92–100. 10.1590/S1809-29502008000100015

[34] Nomura K, Vitti M, de Oliveira AS et al (2007) Use of the Fonseca’s questionnaire to assess the prevalence and severity of temporomandibular disorders in brazilian dental undergraduates. Braz Dent J 18(2):163–167. 10.1590/S0103-64402007000200015 17982559 10.1590/s0103-64402007000200015

[35] Topuz MF, Oghan F, Ceyhan A et al (2023) Assessment of the severity of temporomandibular disorders in females: Validity and reliability of the Fonseca anamnestic index. CRANIO^®^ 41(1):84–87. 10.1080/08869634.2020.1814652 32870743 10.1080/08869634.2020.1814652

[36] Maroco J, Garcia-Marques T (2006) Qual a fiabilidade do alfa de Cronbach? Questões antigas e soluções modernas? Laboratório de Psicologia 4(1):65–90. 10.14417/lp.763

[37] Chew PKH, Dillon DB, Swinbourne AL (2018) An examination of the internal consistency and structure of the Statistical Anxiety Rating Scale (STARS). PLoS ONE 13(3):e0194195. 10.1371/JOURNAL.PONE.0194195 29538400 10.1371/journal.pone.0194195 PMC5851613

[38] Barten JA, Pisters MF, Huisman PA, Takken T, Veenhof C (2012) Measurement properties of patient-specific instruments measuring physical function. J Clin Epidemiol 65(6):590–601. 10.1016/j.jclinepi.2011.12.005 22459427 10.1016/j.jclinepi.2011.12.005

[39] Streiner DL, Norman GR (2008) Health Measurement Scales. Oxford University Press. 10.1093/acprof:oso/9780199231881.001.0001

