# Supplementary material for: Portuguese translation, cultural adaptation and psychometric properties of the temporomandibular joint scale: a cross-sectional study
Source: Oral Maxillofac Surg. 2024 Oct 31;29(1):3. doi: 10.1007/s10006-024-01300-8 (PMC11527962; doi:10.1007/s10006-024-01300-8)
Supplement: Supplementary file 1 — Supplementary Material 1 [file 10006_2024_1300_MOESM1_ESM.pdf]

Nome do Clínico ______________________

Morada ______________________

____

TMJ SCALE™

Este questionário é projetado para ajudar o seu clínico a avaliar o seu problema. Por favor responda a todas as questões da forma mais honesta possível. Use um **lápis N 2 preto.** Marque as respostas claramente, apagando completamente quaisquer mudanças. Não faça marcas fora dos espaços das respostas. **Não deixe nada por responder,** mesmo que não tenha certeza absoluta. (Exemplo de marcação)

Iniciais:__________ Data de hoje___/___/___ Ficha No. (preenchido pelo clínico) ______ __________

Idade _____ Sexo (marque um) [1] Masculino [2] Feminino

Estado Civil (marque um) [1] Solteiro(a) [2] Casado(a) [3] Separado(a) [4] Divorciado(a) [5] Viúvo(a) [6] Re-casado(a)

Etnia/ Grupo Racial (marque um) [1] Negro [2] Hispânico [3] Asiático [4] Branco [5] Outro

Número de anos escolares (marque um) [1] [2] [3] [4] [5] [6] [7] [8] [9] [10] [11] [12] [13] [14] [15] [16] [17] [18] [19] [20+]

Duração do Problema (marque um) [1] Nada [2] Menos de 1 Mês [3] 1-5 Meses [4] 6-11 Meses [5] 1-2 Anos [6] 3-5 Anos [7] 6-10 Anos [8] 10+ Anos

1. Esta pergunta deve só ser respondida se tiver dentes frontais superiores ou inferiores, ou se estiver a usar um substituto para eles. Abra a boca o mais possível e posicione a sua mão como demonstrado no diagrama abaixo.
Coloque o maior número de dedos possível entre os dentes da frente de cima e debaixo. Agora **marque um número** em baixo indicando o **número de dedos.**

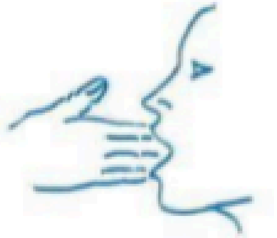

(marque um)

Menos de 1 dedo.......... [0]
Pelo menos 1 dedo.......... [1]
Pelo menos 2 dedos.......... [2]
Pelo menos 3 dedos.......... [3]
Pelo menos 4 dedos.......... [4]

Para as perguntas #2-8 em baixo, localize cada área do seu rosto (excepto F) usando o diagrama de letras. Pressione cada área firmemente em ambos os lados do seu rosto. **Marque o número** que indica o **máximo de dor** que sente.

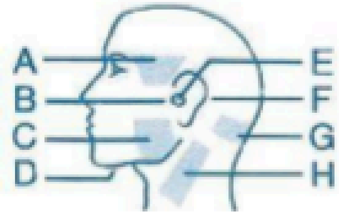

Sem dor 0
Dor ligeira 1
Dor moderada 2
Dor forte 3
Dor Extrema 4

(marque um)

2. Pressionando as minhas têmporas (A no diagrama).......... [0] [1] [2] [3] [4]
3. Pressionando as articulações da mandíbula (B no diagrama).......... [0] [1] [2] [3] [4]
4. Pressionando os músculos da mandíbula (C no diagrama).......... [0] [1] [2] [3] [4]
5. Pressionando os músculos abaixo e lateralmente à mandíbula (D no diagrama) [0] [1] [2] [3] [4]
6. Pressionando nos ouvidos (E no diagrama).......... [0] [1] [2] [3] [4]
7. Pressionando atrás do pescoço (G no diagrama).......... [0] [1] [2] [3] [4]
8. Pressionando os dois lados do pescoço (H no diagrama).......... [0] [1] [2] [3] [4]

S.R. Levitt M.D., Ph.D., T.F. Lundeen, D.M.D., M.W. McKinney, Ph.D.
Copyright ©2018 Pain Resource Center, Inc. All Rights Reserved.

Assinale o número que melhor descreve **quantas vezes** cada afirmação listada abaixo se aplica a si, usando a seguinte chave:

Nunca	**0**
Às vezes	**1**
De vez em quando	**2**
Bastantes vezes	**3**
Sempre	**4**

		(marque um)
**9.**	Um pequeno toque na minha face causa-me uma dor tipo choque.………...	[0] [1] [2] [3] [4
**10.**	A minha mandíbula tem de clicar ou estalar antes de conseguir abrir bem....	[0] [1] [2] [3] [4
**11.**	A minha mandíbula abre completamente sem movimentos laterais…………	[0] [1] [2] [3] [4
**12.**	A minha mandíbula fica bloqueada quando abro…………...…………....	[0] [1] [2] [3] [4
**13.**	Tenho dores de cabeça que começam depois de ver luzes a piscar ou manchas escuras.......	[0] [1] [2] [3] [4
**14.**	A minha mandíbula move-se com facilidade.…………………………………	[0] [1] [2] [3] [4
**15.**	Tenho problemas de saúde que não responderam a nenhum tratamento.....	[0] [1] [2] [3] [4
**16.**	Tenho dor nas articulações da mandíbula (B no diagrama)………………...	[0] [1] [2] [3] [4
**17.**	A minha mandíbula cansa-se facilmente quando mastigo…………………...	[0] [1] [2] [3] [4
**18.**	Tenho dores de cabeça que agravam com a luz intensa……...………..........	[0] [1] [2] [3] [4
**19.**	Doem-me os dentes quando mordo……………………...…………….	[0] [1] [2] [3] [4
**20.**	Tenho dores musculares ou articulares noutras áreas para além da cabeça ou pescoço...	[0] [1] [2] [3] [4
**21.**	Consigo mover a minha mandíbula mais para um lado do que para o outro…	[0] [1] [2] [3] [4
**22.**	Sinto-me tenso(a) ou preocupado(a)………………………………………	[0] [1] [2] [3] [4
**23.**	Tenho perda de líquido pelo(s) meu(s) ouvido(s)…………………………	[0] [1] [2] [3] [4
**24.**	Sinto-me triste ou deprimído………………………………………………	[0] [1] [2] [3] [4
**25.**	Cerro os meus dentes……………………………………………………..	[0] [1] [2] [3] [4
**26.**	A minha mordida é confortável....…………………………………………	[0] [1] [2] [3] [4
**27.**	Tenho dores na mandíbula que pioram com o movimento……………...….	[0] [1] [2] [3] [4
**28.**	É difícil encontrar uma posição confortável para a minha mandíbula..……..	[0] [1] [2] [3] [4
**29.**	Tenho dor de ouvido(s) (E no diagrama).………………………………..	[0] [1] [2] [3] [4
**30.**	Tenho problemas de sinusite...……………………………………………...	[0] [1] [2] [3] [4
**31.**	Quando mordo normalmente, os dentes da frente tocam-se………………....	[0] [1] [2] [3] [4
**32.**	Durante a minha vida, tenho tido várias condições clínicas que me causaram dor...	[0] [1] [2] [3] [4
**33.**	Tenho dor na face que surge de repente como choques eléctricos....…….	[0] [1] [2] [3] [4
**34.**	Consigo abrir a minha boca ao máximo e sem dor…......…………………...	[0] [1] [2] [3] [4
**35.**	Tenho dor dentro ou por detrás do(s) olho(s)……………………………...	[0] [1] [2] [3] [4
**36.**	A minha mandíbula range quando abro e fecho a boca ……………...…….	[0] [1] [2] [3] [4
**37.**	Acho que a posição da minha mandíbula está errada....……………………	[0] [1] [2] [3] [4
**38.**	A minha dor fica pior com stress ou tensão…………………………......	[0] [1] [2] [3] [4

Assinale o número que melhor descreve **quantas vezes** cada afirmação listada abaixo se aplica a si, usando a seguinte chave:

Nunca	**0**
Às vezes	**1**
De vez em quando	**2**
Bastantes vezes	**3**
Sempre	**4**

(marque um)

39. A minha mandíbula clica ou estala quando mastigo............................………. [0] [1] [2] [3] [4

40. Consigo morder com força sem dor na mandíbula…..………………………... [0] [1] [2] [3] [4

41. Um problema doloroso é seguido por outro………………………………… [0] [1] [2] [3] [4

42. Tenho dor na mandíbula que me fazem sentir doente e febril…..………….. [0] [1] [2] [3] [4

43. Ranjo os dentes durante o dia………………………………………………… [0] [1] [2] [3] [4

44. Tenho áreas dormentes na minha face...…………………………………….. [0] [1] [2] [3] [4

45. Uso medicação para os nervos, para dormir, ou álcool para aliviar………… [0] [1] [2] [3] [4

46. Consigo mover a minha mandíbula facilmente……………………………... [0] [1] [2] [3] [4

47. Consigo mastigar sem bater os dentes inesperadamente.…………………... [0] [1] [2] [3] [4

48. Sinto formigueiro na face..................………….............…………………….. [0] [1] [2] [3] [4

49. Tenho dor nos músculos da mandíbula (C no diagrama)…......…………… [0] [1] [2] [3] [4

50. Tenho dor na parte na parte de trás do pescoço (G no diagrama)…...........….. [0] [1] [2] [3] [4

51. Ao longo dos anos, tenho estado sob muito stress……..............……….. [0] [1] [2] [3] [4

52. A minha mandíbula tem contrações musculares incontroláveis....…………….. [0] [1] [2] [3] [4

53. Quando mordo normalmente, os meus dentes de trás tocam-se……………... [0] [1] [2] [3] [4

54. A forma como os meus dentes da frente se encaixam parece estar a mudar.... [0] [1] [2] [3] [4

55. Um toque leve num dos lados da minha face causa dor tipo choque do outro lado.. [0] [1] [2] [3] [4

56. Tenho zumbido no(s) meu(s) ouvido(s)..……………………………………… [0] [1] [2] [3] [4

57. Tenho dor que fica pior com certas pessoas ou situações……………... [0] [1] [2] [3] [4

58. Tenho dor no(s) lado(s) do meu pescoço (H no diagrama)………………… [0] [1] [2] [3] [4

59. Tenho regularmente dor de cabeça ……………………………...………. [0] [1] [2] [3] [4

60. Tenho muita dor que está sempre a mudar……………………………. [0] [1] [2] [3] [4

61. Sinto-me zangado(a)…………………………………………………………... [0] [1] [2] [3] [4

62. Outras pessoas notam ruído na minha mandíbula quando mastigo...……….. [0] [1] [2] [3] [4

63. Consigo mastigar comida tão bem como sempre……………………………… [0] [1] [2] [3] [4

64. Tenho problemas de saúde que parecem estar a piorar...............……………. [0] [1] [2] [3] [4

65. Tenho dor nos músculos por baixo da minha mandíbula (D no diagrama)..... [0] [1] [2] [3] [4

66. Tenho dor nas minhas tempôras (A no diagrama)....………………………… [0] [1] [2] [3] [4

67. Sinto-me ansioso(a)……………………………………………………………… [0] [1] [2] [3] [4

68. Consigo abrir a boca tão bem como costumava.......………………………… [0] [1] [2] [3] [4

Assinale o número que melhor descreve **quantas vezes** cada afirmação listada abaixo se aplica a si, usando a seguinte chave:

Nunca	**0**
Às vezes	**1**
De vez em quando	**2**
Bastantes vezes	**3**
Sempre	**4**

(marque um)

69. A forma como os meus dentes de trás se encaixam parece estar a mudar….. [0] [1] [2] [3] [4

70. Durmo bem………………………………………………………………….. [0] [1] [2] [3] [4

71. Tenho dores de cabeça ou faciais que ficam piores quando me curvo.........… [0] [1] [2] [3] [4

72. Quando toco um lado da minha face, o outro lado fica dormente..………... [0] [1] [2] [3] [4

73. A minha mandíbula fica presa e não abre muito…………………………… [0] [1] [2] [3] [4

74. Os únicos problemas na minha vida são os da minha saúde física…... [0] [1] [2] [3] [4

75. Tive diferentes opiniões entre diferentes clínicos sobre os meus problemas de saúde [0] [1] [2] [3] [4

76. Consigo mover a minha mandíbula em qualquer direcção sem dores……… [0] [1] [2] [3] [4

77. Tenho dor na face que fica pior com o tempo frio.................…………… [0] [1] [2] [3] [4

78. Sinto-me frustrado(a)……………………………………………………. [0] [1] [2] [3] [4

79. Tenho o nariz entupido…………………………………………………... [0] [1] [2] [3] [4

80. Tenho estado sob muito stress recentemente……..……………………. [0] [1] [2] [3] [4

81. Tenho dor de cabeça que me faz sentir enjoado(a)........……………… [0] [1] [2] [3] [4

82. Consigo dar trincas a coisas grandes como maçãs..………………………... [0] [1] [2] [3] [4

83. Sinto pressão familiar ou profissional………………………………..... [0] [1] [2] [3] [4

84. Tenho dor e rigidez nas articulações dos dedos…………………..…… [0] [1] [2] [3] [4

85. Os dentes de trás parecem que se encaixam normalmente………………….. [0] [1] [2] [3] [4

86. Acredito ter um problema incurável apesar da garantia clínica do contrário.. [0] [1] [2] [3] [4

87. De manhã os meus dentes estão doridos e sinto a minha mandíbula cansada.. [0] [1] [2] [3] [4

88. Os meus ouvidos sentem-se entupidos ou bloqueados.........................……...... [0] [1] [2] [3] [4

89. Tenho vários problemas de saúde…………………………………..…….. [0] [1] [2] [3] [4]

90. A minha mandíbula move-se para a frente como sempre...............………. [0] [1] [2] [3] [4]

91. Tenho dificuldade em engolir……………………………………….……. [0] [1] [2] [3] [4]

92. Tenho dor atrás do(s) ouvido(s) (F no diagrama)……………………... [0] [1] [2] [3] [4]

93. Tenho dor na face quando outras articulações também estão doridas……... [0] [1] [2] [3] [4]

94. Tenho problemas nervosos…………………………………………………. [0] [1] [2] [3] [4]

95. Tenho dor de cabeça latejante…………………………………………….. [0] [1] [2] [3] [4]

96. Sinto-me tonto(a)………………………………………………….……… [0] [1] [2] [3] [4]

97. Considero-me uma pessoa doente……………………………………… [0] [1] [2] [3] [4]
