# Supplementary material for: Portuguese translation, cultural adaptation and psychometric properties of the temporomandibular joint scale: a cross-sectional study
Source: Oral Maxillofac Surg. 2024 Oct 31;29(1):3. doi: 10.1007/s10006-024-01300-8 (PMC11527962; doi:10.1007/s10006-024-01300-8)
Supplement: Supplementary file 2 — Supplementary Material 2 [file 10006_2024_1300_MOESM2_ESM.docx]

Abbreviations

BMI - body mass index

CI - confidence interval

COSMIN - Consensus-based Standards for the selection of health Measurement Instruments

ICC - intraclass correlation coefficient

PROMs – Patient-Report Outocomes Measures

SPSS - Statistical Package for Social Sciences

TMJ - temporomandibular joint

TMD - temporomandibular joint disorders

UFP - Fernando Pessoa University
